# Study on the dynamic prediction and optimum regulation scheme of water resource carrying capacity in the yellow river basin

**DOI:** 10.1038/s41598-025-93427-1

**Published:** 2025-03-10

**Authors:** Ke Zhou

**Affiliations:** North China University of Water Resources and Electric Power, Jinshui Road 136, Zhengzhou, 450046 Henan China

**Keywords:** WRCC, Dynamic prediction, Models and methods, Optimum regulation, Yellow river basin, Hydrology, Limnology, Materials science

## Abstract

The dynamic change in regional water resource carrying capacity (WRCC) is an important factor in formulating an optimal water resource allocation scheme. To accurately predict the dynamic change in WRCC and formulate the optimal regulation scheme in a river basin, this paper proposes a diagnostic index system of WRCC based on the mutual feeding mechanism of economic-society, water resources and eco-environment. The multi-methods including support vector machine (SV) and system dynamics (SD), back propagation neural network (BP), etc., were used to construct a quantitative dynamic prediction model for the WRCC. The orthogonal test (OT) method is used to optimize the WRCC regulation scheme. Through simulation, the WRCC in the sub-regions of the Yellow River Basin in 2030, 2035 and 2050 under different scenarios are obtained. The study results show that the WRCC in the upper, middle and lower reaches of the Yellow River Basin are overloaded or seriously overloaded before the operation of the Western Line of South-to-North Water Transfer Project (WLSNWTP). Six key driving indexes (unconventional water utilization, water flow into the sea, irrigation water use, irrigation area, industrial added value and water consumption of 10 thousand Yuan (CNY) industrial added value) are selected to carry out orthogonal tests, and the optimal WRCC regulation scheme is obtained in different level years. The present study results have an important reference value for efficient water resource utilization and eco-society development in the Yellow River Basin.

## Introduction

In recent years, the swift advancement of economic and societal development, coupled with a rise in water consumption, has led to severe issues concerning water resource availability, environmental capacity, and ecological space globally. In certain regions, the exploitation of water resources has surpassed their sustainable carrying capacity. Consequently, the primary concerns in water resource security have transitioned from a scarcity of water supply to an excess burden on water resource capacities^1–3^.

There are three main viewpoints to depict WRCC: the first is the water resource development scale; the second is water resource sustainable development; and the third is the largest population supported by water resources. At present, research on WRCC is mostly inclined to evaluate its grade or status, including the determination of index weight and prediction. However, there are few studies on the diagnosis or regulation of WRCC indexes^4–6^.

In recent years, most WRCC studies have been focused on the concepts of sustainable water resource utilization and water resource restriction. Dehaghi et al. (2020) studied the optimal allocation of water reuse by using a modified Todim-Gp approach^7^. Kicsiny and Varga (2019) set up a differential game model for limited water resources^8^. Nasiri-Gheidari et al. (2018) proposed a robust multi-objective bargaining methodology for inter-basin water resource allocation^9^. Pourmand, et al. (2020) proposed a multi-criteria group decision-making methodology using interval Type-2 fuzzy sets and applied it to water resource management^10^. Sarband, et al. (2020) developed an interactive spatial multi-attribute decision support system for assessing water resource allocation scenarios^11^. Mehrazar, et al. (2020) applied the adaptation of a water resource system to water scarcity and climate change in the suburban area of megacities^12^. Mirdashtvan, et al. (2021) studied sustainable water supply and demand management in semiarid regions^13^.

In China, Academician WANG, et al. (2004) studied national water resource allocation for 30 years based on high efficiency water use targets and low-carbon emissions with the multidimensional decision-making mechanism and predicted the future development direction of comprehensive water resource demand^14^. JIN, et al. (2018) used the improved differential coefficient distribution method to study the WRCC of Anhui Province^15^. Song, et al. (2011) used fuzzy analysis and a neural network model to evaluate and predict the WRCC in Tianjin^16^. ZUO (2017) carried out a comprehensive study on the WRCC evaluation method and achieved some constructive conclusions^17^. Hossein Yousefi and Ali MoridiHossein (2022) studied multi-objective optimization of agricultural planning considering climate change impacts on Minab reservoir upstream watershed in Iran^18^. The study developed a simulation-optimization model by integrating the Soil and Water Assessment Tool (SWAT) and the Non-Dominated Sorting Differential Evolution (NSDE) algorithm. The SWAT model is used to simulate the hydrological and agricultural processes in the watershed, while the NSDE algorithm is employed to optimize the agricultural planning strategies. Hasan Nazari Mejdar, et al. studied water quantity–quality assessment in the transboundary river basin under climate change: a case study^19^. The study combined hydrological and water quality simulation models with a water resource planning model. The Soil and Water Assessment Tool (SWAT) and Water Evaluation and Planning (WEAP) model were used to simulate and evaluate the water quantity and quality in the Doosti Dam Basin on the Harirud River. Five bias-corrected climate models were applied based on Representative Concentration Pathway (RCP) scenarios to project future climate conditions.

Considering the studies above, the current research findings on WRCC both in China and abroad have the following problems. First, the input and output of natural water resources, economic development and spatial characteristics, the spatial characteristics of water resources, vary across different regions. This variability makes it impossible to directly apply research findings from one region to another. Second, the dynamic change in water resource conditions results in different water resource carrying capacities, in which the influencing factors and features are varied. Third, most of the previous studies focus on the current water resource and social economic conditions, which could obtain the current WRCC. Such results could not determine the WRCC under the conditions of future climate and water resource changes^20^. Therefore, it is necessary to propose a systematic method with multidimensional comprehensive water resource evaluation and accurate identification of regional WRCC according to the dynamic changes in regional water resource conditions. A multidimensional comprehensive evaluation of regional WRCC is the premise for accurately identifying water resource dilemmas and the basis for formulating different regional water resource development and utilization policies, which has an important reference value for improving the development and utilization of water resources in a river basin.

In this paper, according to WLSNWTP construction target, taking the maximum water resources development scale as the research objective, this study delves into the water resource carrying capacity in terms of quantity, quality, spatial distribution, and flow, aiming to optimize the corresponding regulatory schemes, including index diagnosis, prediction and regulation. The demonstration was carried out in the Yellow River Basin.

## Methodologies

### Construction of the water resources carrying capacity diagnosis system

#### Mechanism analysis of water resource carrying capacity

**Fig. 1:**
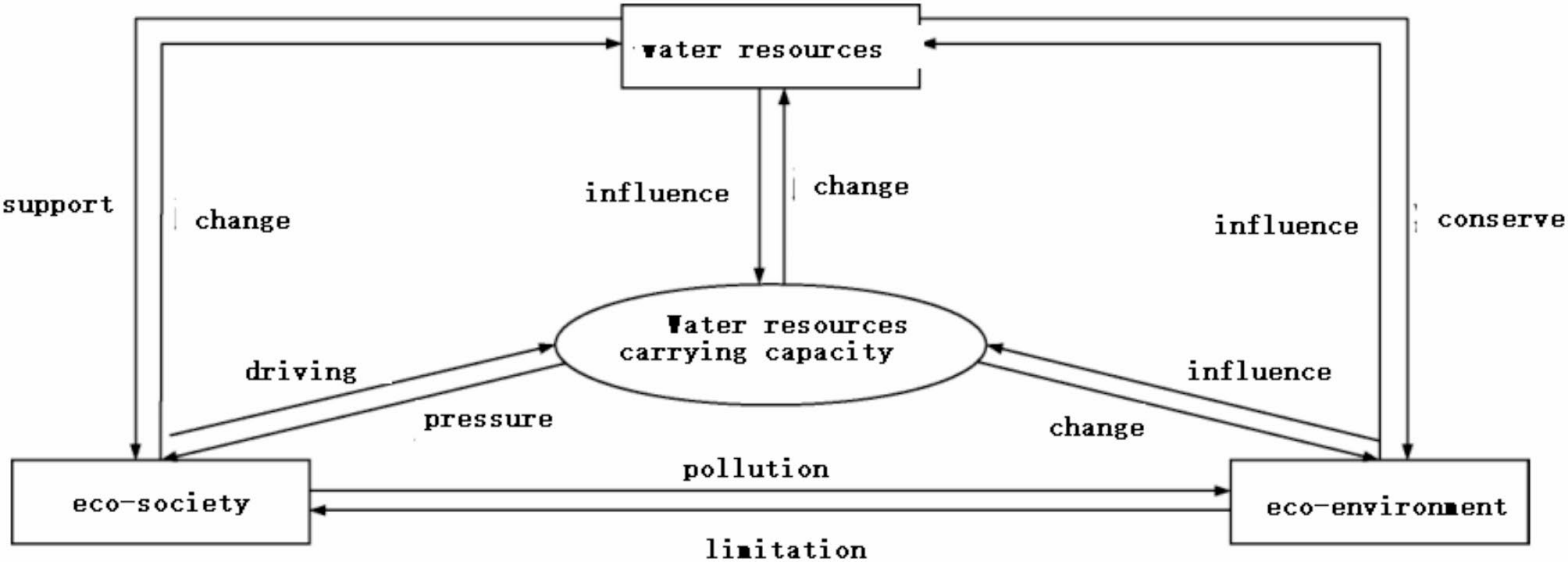
The WRCC constraints by social economy, water resources and eco-environment.

The WRCC is constrained by three systems, as shown in Fig. 1. The water resources system directly supports the eco-society and protects the eco-environment system. Water resource generation, migration, consumption and discharge in the water circulation process are closely related to the WRCC. The contradiction between water resource supply and demand caused by eco-society development is a direct pressure on WRCC. The water resource quantity could change with the development of society and the economy. Thus, water shortages could occur. Water resource quality could also be changed by human activities through pollutant emissions, which result in water quality deterioration and cause water area spatial patterns and water flow renewable process variations^21,22^.

The influence of four-dimensional elements (water quantity, quality, space and flow) on the water resources carrying capacity could be reflected in the following aspects:

- **Water quantity**. The maximum water consumption amount in a region (the total available surface and underground water quantity) is mainly limited by the following two factors: ① the regional water resource recycling and renewable capacity (such as annual runoff, recharge and renewable water resources); ② the eco-environmental water demand, including water demand in rivers, lakes, underground water space, and the different water function zones to maintain health lives in the different water bodies.
- **Water quality**. The maximum water environmental capacity in a region refers to the maximum allowable pollution water drainage amount threshold, which is closely related to the water circulation state and the water self-purification capacity in the region. Water quality protection objectives have two requirements: ① to meet the requirements of water function zone planning and ② to protect water ecosystem security and biodiversity.
- **Water space.** The maximum allowable development of water space, rivers and riparian lakes in a region could be considered the basic factors for water ecosystem health maintenance. The WRCC within the water space dimension is mainly reflected as providing suitable space for health lives (or ecological systems) in rivers, lakes and wetlands. Water space utilization and relevant influences should be controlled within a reasonable scope to reserve living habitat space for aquatic organisms and migratory birds and provide a necessary physical basis for regional water circulation systems and natural water purification in water bodies.
- **Water flow.** The maximum allowable disturbance range for river flow and lake water body refers to the threshold of flow barrier degree or allowable flow variation degree, which includes two aspects, ① the threshold of the water system connectivity degree (longitudinal, transverse and vertical) and ② the threshold of flow rate and flow status index.

#### Water resource carrying capacity evaluation system

WRCC can be classified as WRCC ability and WRCC load. This paper analyzed the WRCC from the perspectives of quantity, quality, domain, flow and natural replenishment, economy and society, and eco-environment. More than 100 indicators were selected. Through correlation analysis, main element analysis, and entropy weight analysis, 24 key driving indicators were screened from the selected indicators in the Yellow River Basin, as shown in Table 1.

**Table 1:** Key driving indicators of WRCC in the yellow river basin.

Element	Indicators	Driving indicators
Water quantity	Regional available water resources Regional water use	Rainfall Total water resources amount Water storage in the large and middle reservoirs Unconventional water consumption Water use efficiency
		Population density Water use ten thousand GDP Water use/ten thousand Yuan industrial added value
Water quality	Sewage drainage capacity Into water function zones (COD, NH_4_+-N) Pollutants into rivers from water function (COD, NH_4_+-N)	River flow Annual runoff Average river discharge Total COD emission amount Total NH_4_+-N emission amount Urban sewage water treatment rate
Water space	The minimum eco-water area	Rainfall Annual runoff River and lake density (remote sensing) Water and soil erosion rate Hydrological connection degree Vegetation coverage rate
	The real water area	
River flow	Annual runoff in different river sections Total reservoir storage regulation	Total water resources quantity Annual average river discharge rainfall
		Annul runoff variation rate Discharge variation Degree
		River pulsation index

### Water resources carrying capacity prediction

#### The scheme of WRCC dynamic prediction

**Fig. 2:**
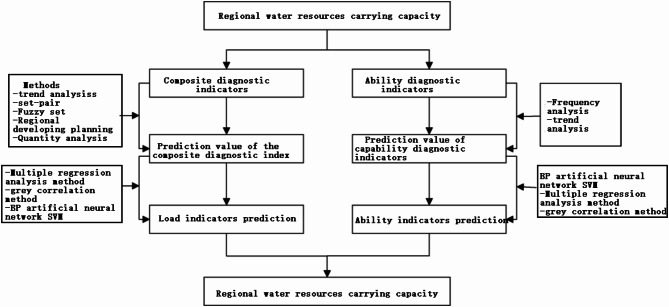
Technical scheme for regional water resource carrying capacity prediction.

Through WRCC diagnostic index analysis, a WRCC diagnostic index prediction model was constructed by using intelligent modeling methods, including artificial neural networks (BP), genetic algorithms (GA), support vector machines (SV), set analysis (SA) and system dynamics (SD) models^23,24^. The relationship between the water resource load and diagnostic index was built. Through diagnostic index prediction, the dynamic prediction for the scheme of regional WRCC was realized, as shown in Fig. 2.

#### Correlation analysis of the WRCC and diagnostic index

The correlation between the WRCC (water quantity, quality, space and flow) index (Y) and the diagnostic index (X={x_1_, x_2_, …, x_n_ }) can be calculated by using the transfer function f, as shown in formula (1):

1 $$\:\text{Y}=\text{f}\left(\text{x}\right)=\text{f}({{\text{d}}_{1}\text{x}}_{1},{{\text{d}}_{2}\text{x}}_{2},\:\cdots\:,{{\text{d}}_{\text{n}}\text{x}}_{\text{n}})$$

where d_i_ refers to the driving indicator for the ith diagnostic index.

### Water resources carrying capacity regulation

#### Regulation objective

In view of the complex water resources problems in the Yellow River Basin, based on the development goal of water resource carrying capacity in the national WRCC evaluation and strategic allocation, the serious overload area of the water resource carrying capacity should be eliminated by 2035, and all the overload area of WRCC should be eliminated before 2050. In this paper, the WRCC regulation was conducted according to the above development target. For each region (provinces, cities, and autonomous regions), a series of specific regulation targets should be set according to the timetable of the general objectives (i.e., the comprehensive water resources plan, water pollution prevention and control action plan, main functional zone plan, ecological control red line, and national territorial space development plan^25–27^. The special regulation objective in a region could be higher than the general objective.

#### Regulation method

The water resources carrying capacity regulation scheme involves many aspects, such as complex influence factors and multilevel regulation schemes. In this paper, an orthogonal test was used to carry out regulation studies, in which the optimal scheme could be quickly obtained with fewer trials, shorter test cycles, and lower test and production costs.

#### Regulation scheme

According to the local WRCC diagnostic results and based on the local driving factors (water quantity, quality, water space and river flow) and regulation objective, the regulation scheme was obtained through calculation and analysis.

The driving factors can be classified into two types, i.e., adjustable and un-adjustable factors. For adjustable factors in the planning year (2030, 2035 and 2050), different scenarios were designed. Several regulation schemes can be generated through orthogonal tests, from which final regulated schemes were optimized to realize the development target in the regions.

#### Regulation measures

To ensure the sustainable development of the water resource system, it is necessary to put forward a series of regulatory measures in two ways (i.e., developing sources and saving water) to solve the problems of water resource shortages, water environmental pollution, water area shrinkage and water flow obstacles in different regions. Specific regulation measures were proposed for regulating factors by using the water resource carrying capacity diagnosis index system, prediction model and regulation methods.

## Water resource carrying capacity dynamic prediction and regulation in the yellow river basin

### Background

The Yellow River is the second longest river in China and has played a crucial role in the country’s history and development. It originates from the Bayan Har Mountains in Qinghai Province and the main channel length is approximately 5,464 km (3,395 miles) before emptying into the Bohai Sea at Shandong Province. The Yellow River basin covers an area of about 795,000 square kilometers, which is roughly one-seventh of China’s total land area. The river passes through nine provinces, including Qinghai, Sichuan, Gansu, Ningxia, Inner Mongolia, Shaanxi, Shanxi, Henan, and Shandong. The population of the Yellow River basin is around 400 million people, which accounts for about one-third of China’s total population. The Yellow River basin plays a crucial role in China’s economy and development. Its population, industry, agriculture, and natural resources are all essential components of the country’s growth and prosperity.

The average annual temperature of the basin is 7.8℃, the annual precipitation is 458 mm, the annual relative humidity is 59.1%, the average annual wind speed is 2.3 m/s, the annual sunshine hours is 2 526.7 h, and the annual average potential evaporation is 943 mm. The water area of the river basin is about 5,343.6 km^2^, accounting for 0.67% of the total area of the Yellow River basin. There are 30 major hydrologic stations along the main course of the Yellow River and its key tributaries. The annual runoff data time series (1952–2018) of three control stations (Lanzhou, Huayuankou and Lijin stations) in the upper, middle and lower reaches of the Yellow River basin were adopted in this paper. Based on the sequence characteristic analysis, different methods are used to calculate the design annual runoff of each station. Lanzhou Hydrological Station and Huayuankou Hydrological Station have annual runoff data of 100 years length from 1922 to 2022, and Lijin Hydrological Station has annual runoff data of 67 year length from 1952 to 2018.

The Yellow River basin faces severe water pollution problems. Many industries in the Yellow River basin, such as petrochemicals, coal chemical industry, papermaking, and metallurgy, discharge a large amount of wastewater. In agricultural production in the Yellow River basin, a large amount of chemical fertilizer and pesticides are used. Excessive use of chemical fertilizers leads to a large amount of nitrogen, phosphorus and other nutrients not being absorbed by crops and entering rivers and lakes along with surface runoff, causing water eutrophication. With the acceleration of urbanization in the Yellow River basin, the discharge of domestic sewage is increasing continuously. The Yellow River basin, especially in the Loess Plateau area, has serious soil erosion. Therefore, it is an urgent task to carry out systematic planning of WRCC.

### Diagnosis of the WRCC index in the yellow river basin

The Yellow River Basin is divided into three research zones: upper, middle and lower reaches. Considering the evaluation results, the water resources carrying capacity in the upper, middle and lower reaches of the Yellow River Basin was overloaded in 2017. In accordance with the scientific, representative and operable principles, Pearson correlation analysis and the gray correlation degree method are used to identify the key driving indexes. In terms of the water resource carrying capacity, the surface water resource index has a great influence on the regional available water resources. In terms of the water resource load, the agricultural water consumption index and its secondary driving index have a great influence on regional water use (see Table 2).

**Table 2:** Driving indexes of WRCC in the yellow river basin.

Regional available water resources (capacity index)		Regional water use (load index)			
Capacity driving index	Correlation	Grade I driving index	Weight	Grade II driving index	Correlation
Surface water	0.997	Agriculture water use	0.74	Irrigation area	0.940
				Population	0.919
				Irrigation water use quota	0.849
Underground water	0.937	Industrial water use	0.14	GDP	0.481
				Population	0.917
				Industrial added value	0.575
				GDP	0.572
Annual reservoir water storage change	0.699	Domestic wateruse	0.09	Water use/10 thousand Yuan industrial added value	0.517
				Rural domestic water use	0.931
				Urban domestic water use	0.898
				Population	0.858
				GDP	0.678
Annual rainfall	0.658	Ecological water use	0.03		

### Prediction of the water resource carrying capacity index in the yellow river basin

Due to climate change and the development of industry and agriculture, the water resource situation in the Yellow River Basin will change greatly in the future. In this paper, two different scenarios (i.e., before and after operation of the Western Line of South-to-North Water Transfer Project, WLSNWTP) were set. Through simulation tests, the water resource carrying capacity indexes in 2030, 2035 and 2050 under different scenarios were obtained.

According to the prediction results, the water resources carrying capacity in the upper, middle and lower reaches of the Yellow River Basin are seriously overloaded before the operation of the WLSNWTP. After operation of the WLSNWTP, the water resources carrying capacity is at critical status in the middle reach in 2030, 2035 and 2050, and the water resources carrying capacities in most reaches are in serious overload status in the planning level years, as shown in Table 3 (in Table 3, S. over refers to serious overloaded, over means overloaded).

**Table 3:** WRCC before and after the WLSNWTP.

Regions	Prediction (years)	Available water resources (10^8^m^3^)		Water use (10^8^m^3^)		Carrying capacity (10^8^m^3^)		Status	
		Before	After	Before	After	Before	After	Before	After
River basin	2030	515.95	610.95	639.96	639.96	1.240	1.048	S. over	Over
	2035	510.21	605.25	645.85	645.85	1.266	1.067	S. over	Over
	2050	506.60	601.60	660.52	660.52	1.304	1.098	S. over	Over
Upper region	2030	198.52	248.52	282.74	282.74	1.424	1.138	S. over	Over
	2035	197.88	247.88	284.02	284.02	1.435	1.146	S. over	Over
	2050	196.45	246.45	288.05	288.05	1.466	1.169	S. over	Over
Middle region	2030	182.70	226.70	221.48	221.48	1.212	0.977	S. over	Critical
	2035	181.58	225.58	234.72	234.72	1.238	0.996	S. over	Critical
	2050	180.54	224.54	230.78	230.78	1.278	1.028	S. over	Critical
Lower region	2030	134.71	135.71	135.75	135.75	1.008	1.000	Over	Critical
	2035	130.75	131.75	137.12	137.12	1.049	1.041	Over	Over
	2050	129.60	130.59	141.69	141.69	1.093	1.085	Over	Over

### Regulation of the water resources carrying capacity index in the yellow river basin

According to the above prediction results, considering the operation and cost of the regulation scheme and based on two scenarios before and after the WLSNWTP, the water resource carrying capacity and load were studied by using orthogonal test methods. The water resource carrying capacity regulation was carried out in the whole basin to eliminate the overload state in the planning level year, i.e., to control the value of the water resource carrying capacity index within 1.

Through sensitivity analysis on the indexes in the system dynamic model, the driving factors affecting the water resources carrying capacity indexes were optimized. To improve the water resources carrying capacity, two indicators (unconventional water utilization and water flow into the sea) were added to realize desirable regulation. To reduce the water resource carrying load, four indexes (irrigation water consumption, irrigation area, water use/10 thousand Yuan industry added value and industry added value) were regulated. Each index was orthogonally tested in three levels to obtain different regulation schemes. The simulation model was operated to obtain the water resources carrying status under different regulation schemes and to realize water resources balance before and after the operation of the WLSNWTP. The optimum scheme is shown in Table 4.

**Table 4:** Regulation scheme of WRCC before and after the WLSNWTP in the yellow river basin.

Scenarios	Prediction (years)	Raising carrying capacity (10^8^m^3^)		Reducing water load (%)				Carrying capacity state value		Carrying capacity state	
		UWU	WFS	IWU	IA	WU10TIAV	IAV	Before regul.	After regul.	Before regul.	After regul.
Before WLSNWTP	2030	+ 20	− 15	− 20	− 10	− 10	− 5	1.240	0.999	S. over	critical
	2035			− 20	− 10	− 15		1.266	0.968	S. over	critical
	2050			− 30	− 15	− 10		1.304	0.944		
After WLSNWTP	2030	+ 20		− 5	− 5	− 5		1.048	0.980		
	2035			− 5		− 5		1.067	0.999		
	2050			− 10		− 15		1.098	0.962		

In this table, UWU refers to unconventional water use; WFS refers to water flow into the sea; IWU refers to irrigation water use per ha; IA refers to irrigation area; WU10TIAV refers to water use/10 thousand Yuan industrial added value; IAV refers to industrial added value; S. over refers to serious overload water resources carrying capacity; and over refers to overload water resources carrying capacity.

The main regulation measures include reducing farmland irrigation area, reducing irrigation water use quota, reducing water consumption per ten thousand yuan (CYN) industrial added value, adjusting industrial structure, increasing unconventional water use, developing water transfer projects (such as the west line south-north water transfer project), and reducing water inflow into the sea in a timely manner.

## Conclusions

- Based on four elements of WRCC (quantity, quality, domain and flow), this paper used the main component analysis and entropy weight methods to set up the water resources carrying capacity diagnosis system from two aspects (capacity and load). Twenty-four key driving indexes were selected in the Yellow River Basin.
- The prediction model of WRCC was constructed by using the trend analysis method. The relationship between the WRCC and diagnostic indexes was established by using the BP artificial neural network method to obtain the annual water resource carrying capacity status in the different planning level years. The regional WRCC regulation objective was set for the overloaded areas. The orthogonal test method was used to optimize the regulation schemes. The regulation measures were put forward for the index regulation in the optimal scheme. The dynamic prediction and regulation system of the regional WRCC was established.
- Taking the Yellow River Basin as an example, the WRCC and regulation schemes were studied. Key driving indexes were identified by using single element linear analysis, Pearson correlation analysis, and gray correlation methods. The systematic dynamic model was used to predict the water resource carrying status at different planning level years. According to the regulation target, the orthogonal test method was adopted to optimize the water resource regulation schemes before and after the operation of the West Line South-to-North Water Transfer Project in the different planning level years. The studied results have an important reference value for the efficient utilization of water resources and high-quality economic and social development in the Yellow River Basin.
- Indicator weight would influence water resources carrying capacity (WRCC).
- A larger indicator weight would create a greater impact on WRCC. It can be observed from study that the water resources sub-system has the greatest impact on WRCC, followed by the ecology sub-system and the economy sub-system, while the influence of the society sub-system is relatively weak. This difference aligns well with real-world logic, although the weights of the four sub-systems are also subject to dynamic changes.
- To assess the robustness of the results obtained in this study, various methods were employed to reevaluate WRCC for each sub-region in the Yellow River Basin. The results indicate that although there are slight differences in WRCC indices among different research methods, the grading remains consistent, which ensures the reliability of the studied findings.
- Recommendations. To promote the coordinated and sustainable development of WRCC in the Yellow River basin, the following recommendations would be put forward:

1. Formulate differentiated water resource governance policies. Considering the regional differences in the basin, it is recommended to develop differentiated water resource management policies. These policies should fully take into account the variations in natural conditions and local policies to enhance coordination and carrying capacity.
2. Improve water resource utilization efficiency. Given the relative water resource shortage, there is a suggestion to focus on improving water resource utilization efficiency through water resource management.
3. Enhance cross-regional coordination in water resource management. It is advisable to strengthen mechanisms for cross-regional coordination in water resource management, promoting more rational geographic distribution and more efficient economic allocation of water resources.
4. Encourage diversified industrial development. To enhance adaptability and resilience, the different regions should be encouraged to develop diversified economic structures. Supporting and guiding diversification in industries can reduce excessive reliance on water resources, promote the optimization and upgrading of industrial structures, and achieve more synergistic development between water resources and the economy.
5. The water pollution reduction strategies in this basin should be carried out, including to carry out strict environmental access standards, to promote cleaner production, to strengthen wastewater treatment, to promote ecological agriculture, to improve domestic water pollution management, to carry out ecological restoration and protection, to strengthen supervision and management, as well as to promote public awareness on water resources protection and reasonable utilization.
6. The future direction/orientation for further research on water resource carrying capacity in the Yellow River basin would be focused on In-depth study of climate change impacts, to refine the calculation methods and models of ecological water demand in different ecosystems, to conduct a detailed analysis of water use efficiency in different industries and sectors, to improve the water resource management system, to explore the application potential of new water treatment technologies and desalination technologies, etc.

## Data Availability

All the data and materials in the current study are available from the corresponding author upon reasonable request.
